# Factors related to the length of stay for major depressive disorder patients in China: A real-world retrospective study

**DOI:** 10.3389/fpubh.2022.892133

**Published:** 2022-07-29

**Authors:** Peng Cheng, Lirong Wang, Lizhi Xu, Ying Zhou, Guangju Zhao, Li Zhang, Weihui Li

**Affiliations:** ^1^Department of Psychiatry, National Clinical Research Center for Mental Disorders, The Second Xiangya Hospital of Central South University, Changsha, China; ^2^Xiangya School of Medicine, Xiangya Hospital, Central South University, Changsha, China

**Keywords:** depression, the length of stay, inpatient management, public mental health, retrospective study, major depressive disorder

## Abstract

**Background:**

As numerous patients with depression have to be hospitalized because of various reasons, the demand far exceeds the limited bed count in the psychiatry department. Controlling the length of stay (LOS) of the patient is gradually being considered an effective method to alleviate this problem. Given the lack of statistical evidence of the LOS of patients with major depressive disorder (MDD) in China and the strain on the limited psychiatric resources, the purpose of our study was to investigate the LOS of patients with MDD among in-patient samples and to analyze related factors of the LOS in China by building a regression model.

**Method:**

The data were exported from the electronic medical record system. A total of three categories of independent variables were enrolled in our study, namely, demographic, clinical, and biochemical. Univariate analysis and binominal regression analysis were applied comprehensively to find the factors related to the LOS among MDD samples. The discrimination accuracy of the model was evaluated by the receiver operating characteristic (ROC) analysis. ROC analysis indicated that the discrimination accuracy of our model was acceptable (AUC = 0.790, 95% CI = 0.714–0.865, *P* < 0.001).

**Result:**

A total of 254 patients were finally brought into analysis after filtering. Regression analysis indicated that abnormal LDL was the only risk factor of long LOS (OR = 3.352, 95% CI = 1.087–10.337, *P* = 0.035) among all the kinds of variables. Notably, in the statistically irrelevant factors of the LOS, the category of anti-depressant drugs [serotonin–norepinephrine reuptake inhibitor (SNRI) or selective serotonin reuptake inhibitor (SSRI)] prescribed to patients with MDD was not associated statistically with the LOS, which was against our initial hypothesis that the LOS of patients with MDD treated with SNRI would vary from that of the patients treated with SSRI.

**Conclusion:**

Up to our knowledge, our research is the first study to show the potential factors related to the LOS from various domains, especially biochemical indexes, and the effect of drugs, among clinical patients with MDD in China. Our results could provide a theoretical reference for efficient psychiatry hospitalization management and prioritization of allocating medical resources. Future studies are required for updating independent variables which are potentially related to the LOS and verifying existing results in a larger sample.

## Introduction

Depression is a prevalent, prolonged, and easily relapsing psychiatric disorder not only around the world but also in China. According to a study on the Global Burden of Disease (GBD) demonstrated that a total of 970 million people worldwide suffer from mental illness, including 260 million people suffering from depression (1). A recent epidemiology survey among the Chinese national-representative sample indicated that the lifetime prevalence of depressive disorders was 6.8%, while the 12-month prevalence was 3.6% (2). Furthermore, depression has always been one of the main causes of non-fatal health loss. Depression ranked 19th among the illnesses in the world with leading disability-adjusted life years (DALYs) in 1990 and 13th in 2019 (3), indicating that the burden of depression was severe and ever-increasing. Concerned about the huge population and the weak but unbalanced development of psychiatry in China, it is essential for Chinese hospitals to rationally allocate medical resources to optimize efficiency with limited labor and material resources.

Several patients with depression had to be hospitalized rather than receiving outpatient treatment to achieve a better treatment effect for the following, but not limited to, reasons: (1) Non-attendance of outpatients: psychiatric patients were much more likely than other medical specialties to miss their appointment, resulting to a higher risk of relapse and readmission (4). (2) Non-adherence to medication: the prevalence of non-adherence among depression patients ranged from 40 to 66.9% (5–7). The non-adherence rate of anti-depressant drugs was 45.9% (3). Risk of suicide: depression was a widely recognized risk factor for suicide (8), as evidenced by the incidence of suicidal ideation among patients with major depressive disorder was about 56–88% (9), and MDD was a significant risk factor of suicidality in China (10). Given China's massive population, coupled with the high prevalence of depression (11), and the relative scarcity of psychiatric workers as compared with other developed countries (12), the Chinese psychiatry medical system was under severe strain. Controlling the length of stay (LOS) of the patient was gradually recognized as an effective approach to address this issue (13). Some previous studies revealed the average LOS of depression patients from various countries, 37.2 days for the Swiss (14), 24.5 days for the Ethiopians (15), 17.4–18.6 days for the Austrians (16), 8.8 days for the South Koreans (17), which varied because of the differences of population, culture, economic development, and other factors. In addition, potential LOS factors could broadly be classified into the following: (1) Demographic variables: age, gender, and marital status (18, 19); (2) Behavior variables: suicide idea or attempt/compulsory hospitalization (20–22); and (3) Clinical variables: frequency of previous admissions, comorbidity with substance use (23, 24). Up to our knowledge, only a couple of similar studies have been conducted in China.

In total, two aspects of the LOS of patients with MDD in China are worth mentioning. At first, Chinese patients are profoundly influenced by the eastern culture, which is far more restrictive than the western culture. A previous community-based survey in China has revealed that stigmatizing beliefs about depression were widespread in the general public (25). The stigma of depression hampered not only the willingness to seek treatment, but also hospitalization compliance, which affected the LOS. As a result, the LOS and its correlates in China may differ significantly from those of other western countries. Second, with the promulgation of China's new Mental Health Law in 2013 (26), the process of diagnosing, curing, hospitalization, and etc., for mental disorders, including MDD, has been systemized and legalized. The voluntary admission principle was particularly emphasized even for patients with MDD with any potential severe risk (i.e., suicide idea), both admission and discharge should still be supervised by the certificated medical department. Compulsory admission was strictly constrained to avoid possible abuse, except for patients recognized by the Chinese Healthcare Commission applying a certified forensic psychiatry assessment, with high risk or behavior of hurting themselves or others (26). Therefore, the LOS of patients with MDD now in China under the context of a series of the new legislation was certainly different from the previous timing when related law was missing.

Moreover, some limitations existed in previous research about LOS. First, few previous studies enrolled patients with a single diagnosis of the depressive disorders instead of patients with various kinds of mental disorders, which might affect the final results of the LOS. Second, biochemical indexes and pharmacological intervention were rarely collected in most previous studies, given their association with the development and prognosis of depression, and then affect the LOS. For biochemical indexes, thyroid-stimulating hormone (TSH), high-density lipoprotein (HDL), and low-density lipoprotein (LDL) were chosen as target biochemical variables to investigate in our research. TSH abnormalities, which affect 3.0–8.5% of the general population and up to 20% of the elderly (27, 28), were linked to depression by regulating the hypothalamic–pituitary–thyroid (HPT) axis (29), as many studies of different samples demonstrated (30, 31). Moreover, TSH abnormalities have been linked to a decreased response to anti-depressants (32, 33), potentially extending the LOS of patients with depressive disorders. A previous study found that hospitalized psychiatric patients with elevated TSH had longer LOS than counterparts with a normal level of TSH (34). Therefore, we also considered that TSH abnormalities were associated with longer LOS in patients with MDD.

In terms of lipoproteins, previous studies suggested that depression pathogenesis may involve HDL and LDL depletion-mediated alterations in central nerve terminal structure and function and then affect the response to serotonin (35, 36), the effect target of mainstream anti-depressants (37). In addition, many studies have linked lipoproteins to an increased risk of suicidal ideations (38, 39), a serious event associated with longer LOS in psychiatric in-patients (21, 40). Thus, the authors speculated that abnormalities in HDL and LDL could lead to a longer LOS in patients with MDD.

In terms of pharmacological intervention, we compared the effects on LOS of two widely applied anti-depressants, serotonin–norepinephrine reuptake inhibitor (SNRI) and selective serotonin reuptake inhibitor (SSRI). These two anti-depressants differed primarily in terms of target symptoms and treatment response. To begin, given that depression is an affective disorder with multiple neurotransmitters hypofunctions [serotonin (5-HT), norepinephrine, dopamine] (41, 42), specific symptoms of depression could be associated with specific neurotransmitters (43, 44). Serotonin may be linked to anxiety, obsessions, and compulsions; whereas norepinephrine might be relevant to alertness and energy, anxiety, attention, and interest in life. So, SNRI could simultaneously inhibit the reuptake of both serotonin and norepinephrine, treating a wider range of symptoms compared with SSRI, which selectively inhibited the reuptake of serotonin. Second, several studies suggested that the onset time of SSRI was longer than that of SNRI (45, 46), which was associated with presynaptic and postsynaptic adaptive mechanisms secondary to reuptake inhibition of SSRI (47). The increase of 5-HT induced by the SSRI is offset by the negative feedback of 5-HT auto-receptors, which reduces the release of 5-HT. Only after inactivating 5-HT auto-receptors after continuous SSRI treatment could the concentration of 5-HT in the synaptic cleft increase continuously, allowing it to exert its anti-depressant effect (48). Besides, previous systematic reviews found that SNRI had a higher remission and response rate for depression patients compared with SSRI (49, 50). Hence, based on the evidence presented earlier, the authors hypothesized that the LOS of patients with MDD treated with SNRI and SSRI differed.

Given the scarcity of statistical evidence of the LOS of patients with MDD in China, and also the strain on the limited psychiatric resources, the goal of our study was to investigate the LOS of patients with MDD among in-patient samples and to analyze related LOS factors in China by developing a regression model. We hypothesized that the findings of this study could serve as a theoretical foundation for future management optimization of the psychiatry department and efficient allocation of mental health services.

## Materials and methods

### Participants and study design

This retrospective research was conducted in the Second Xiangya Hospital of Central South University, which is one of the four mental health centers in China. Data of patients with MDD were exported from the clinical electronic medical record system. Inclusion criteria were as follows: (1) patients were diagnosed with MDD according to the DSM-V criteria (51); (2) patients were admitted voluntarily following China Mental Health Law and relevant rules of the hospital. Exclusion criteria were as follows: (1) patients with comorbidity of any other mental disorder; (2) any category of patients' data were missing. The period set for data collection was between 2012 and 2020. Our study was proved by the ethnic committee of the Second Xiangya Hospital of Central South University (No.2019204).

A total of 3 categories of independent variables were employed in our study, namely, demographic variables (gender, age, job status, marital status, and ethnicity), clinical variables (previous times of admission and category of anti-depressant drugs), and biochemical variables (TSH, HDL, and LDL). Because of the skew distribution observation in the original data of LOS, the LOS was recorded as a binary variable. This methodological approach has been applied by many studies not only to avoid statistical bias but also a more clinically significant interpretation of results (52). We set <28 days as “Short LOS” and ≥28 days as “Long LOS.” This timing point was selected because of the following reasons: (1) LOS which was more than 28 days often has an adverse effect on patients' salaries because many companies consider 28 days as the limit of temporary leave; (2) 28 days was a common limitation of LOS applied by many Chinese hospitals, to meet relevant rules the medical insurance department stipulated to restrain the spending. Details of the variables are shown in Table 1.

**Table 1:** Descriptive statistics and univariate analysis of variables related to the LOS.

**Variables**	***N*** **(%)**	**Short LOS**	**Long LOS**	χ^2^	***P***
**Demographic variables**					
Gender					
Male	94 (37.0%)	88 (93.6%)	6 (6.4%)	2.013	0.156
Female	160 (63.0%)	141 (88.1%)	19 (11.9%)		
Age					
<18	122 (48.0%)	113 (92.6%)	9 (7.4%)	1.608	0.205
≥18	132 (52.0%)	116 (87.9%)	16 (12.1%)		
Job status					
Unemployed	100 (39.4%)	87 (87.0%)	13 (13.0%)	1.853	0.173
Employed	154 (60.6%)	142 (92.2%)	12 (7.8%)		
Marriage status					
Single, separated, divorced, or widowed	188 (74.0%)	174 (92.6%)	14 (7.4%)	4.680	0.031
Married	66 (26.0%)	55 (83.3%)	11 (16.7%)		
Nationality					
Ethnic Han	249 (98.0%)	225 (90.4%)	24 (9.6%)		0.407^*^
Ethnic minority	5 (2.0%)	4 (80.0%)	1 (20.0%)		
**Clinical variables**					
Frequency of the previous admission					
0	227 (89.4%)	205 (90.3%)	22 (9.7%)		0.737^*^
≥1	27 (10.6%)	24 (88.9%)	3 (11.1%)		
Category of anti-depressant drugs					
SSRI	147 (57.9%)	138 (93.9%)	9 (6.1%)	5.442	0.020
SNRI	107 (42.1%)	91 (85.0%)	16 (15.0%)		
**Biochemical variables**					
*Thyroid function*					
Thyroid Stimulating Hormone (TSH)					
Abnormal	15 (5.9%)	14 (93.3%)	1 (6.7%)		1.000^*^
Normal	239 (94.1%)	215 (90.0%)	24 (10.0%)		
*Lipoprotein Cholesterol*					
High-density lipoprotein cholesterol (HDL-C)					
Abnormal	61 (24.0%)	53 (86.9%)	8 (13.1%)		0.325
Normal	193 (76.0%)	176 (91.2%)	17 (8.8%)		
Low-density Lipoprotein cholesterol (LDL-C)					
Abnormal	34 (13.4%)	26 (76.5%)	8 (23.5%)		0.009^*^
Normal	220 (86.6%)	203 (92.3%)	17 (7.7%)		

* Fisher exact test was conducted.

### Measures

#### Demographic variables

Age group, gender (woman or man), job status (unemployed or employed), marital status (unmarried situations and married), and ethnicity (ethnic Han and ethnic minority). All the demographic information mentioned earlier was collected at the beginning of hospitalization.

#### Clinical variables

Clinical data were recorded during the hospitalization of patients with MDD, including frequency of previous admissions (0 or ≥1) and category of anti-depressant drugs (SNRI or SSRI). We filtered the raw data to just enroll patients who separately used either SNRI or SSNRI into our sample.

#### Biochemical variables

Biochemical variables, including TSH, HDL, and LDL, were also measured based on the blood sample obtained at the beginning of hospitalization. All the biochemical indexes were set as dichotomous variables.

### Statistical analysis

Univariate analyses, including Chi-square analysis and Fisher's exact, were conducted to initially analyze the difference in LOS across demographic, clinical, and biochemical variables. Next, a binomial logistic regression model was built, including various variables as potential factors to further investigate independent factors associated with the LOS among patients with MDD. Factors with statistical significance of *p* < 0.05 were considered. Receiver-operating characteristic (ROC) analyses were conducted to evaluate the accuracy of judging the LOS of patients with MDD of this regression model. The data were analyzed with SPSS, version 25.0 (IBM Corp., New York, USA).

## Results

### Descriptive statistics and univariate analysis

**Figure 1:**
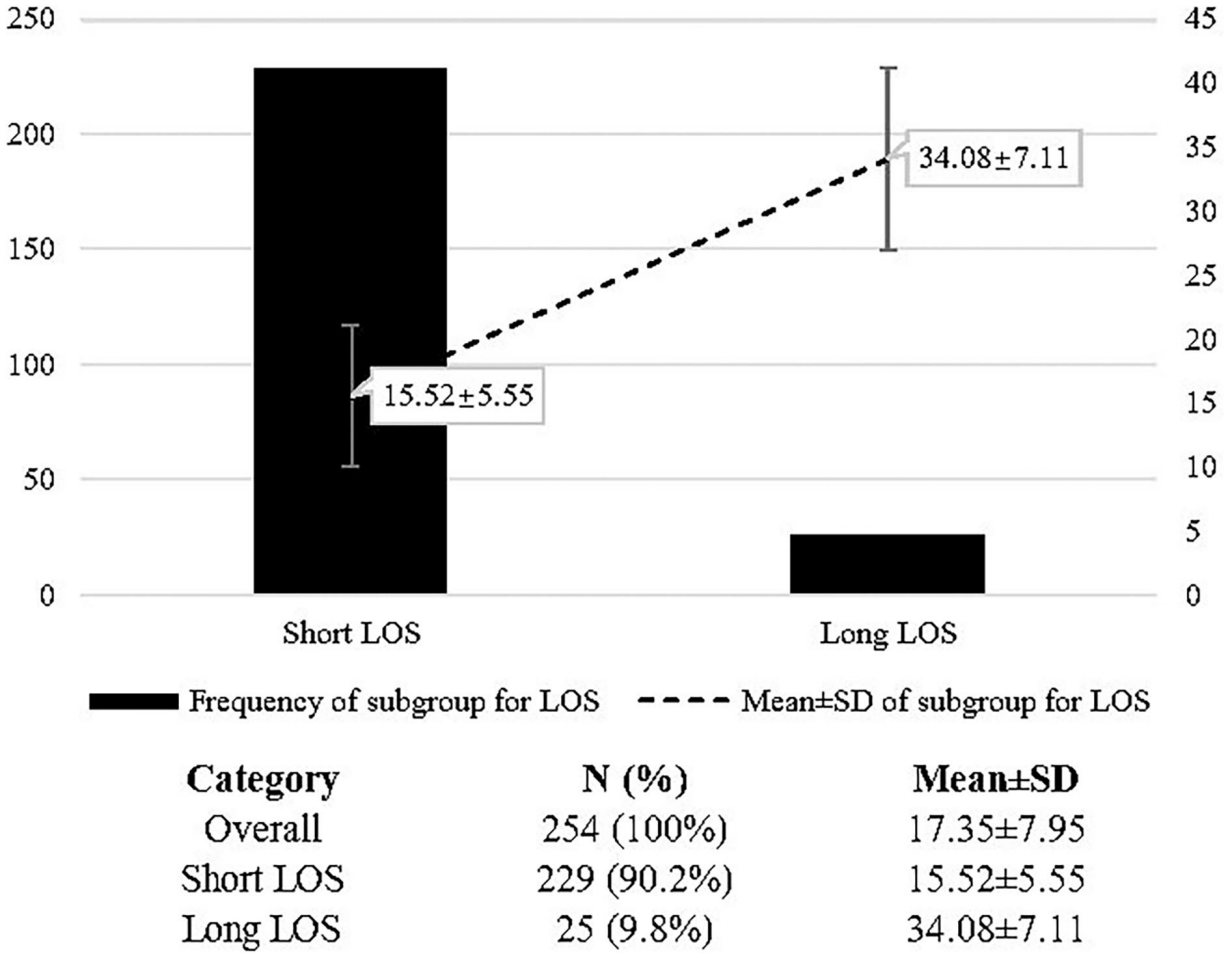
Frequency and mean value of the length of stay (LOS) for the subgroups.

A total of 620 patients' raw information was exported from the electronic medical system and 254 patients were finally brought into analysis after filtering. In total, 229 patients were enrolled for the short LOS group while 25 patients were enrolled for the long LOS group. The mean LOS of the long LOS group (34.08 ± 7.11) was much more than that of the short LOS group (15.52 ± 5.55). Details seen in Figure 1.

The majority of the sample were of women (63.0%), adults (≥18 years old: 52.0%), employed (60.6%), single, separated, divorced, or widowed (74.0%), and ethnic Han (98.0%). Univariate analysis demonstrated the difference of the LOS across variables. The LOS was significantly different on the variables, including marriage status (*P* = 0.031), category of anti-depressant drugs (*P* = 0.020), and LDL (*P* = 0.009). Patients with MDD who were married, using SNRI anti-depressant drugs, and with abnormal LDL, were more likely to have long LOS. Details are in Table 1.

### Binominal regression analysis and ROC analysis

**Figure 2:**
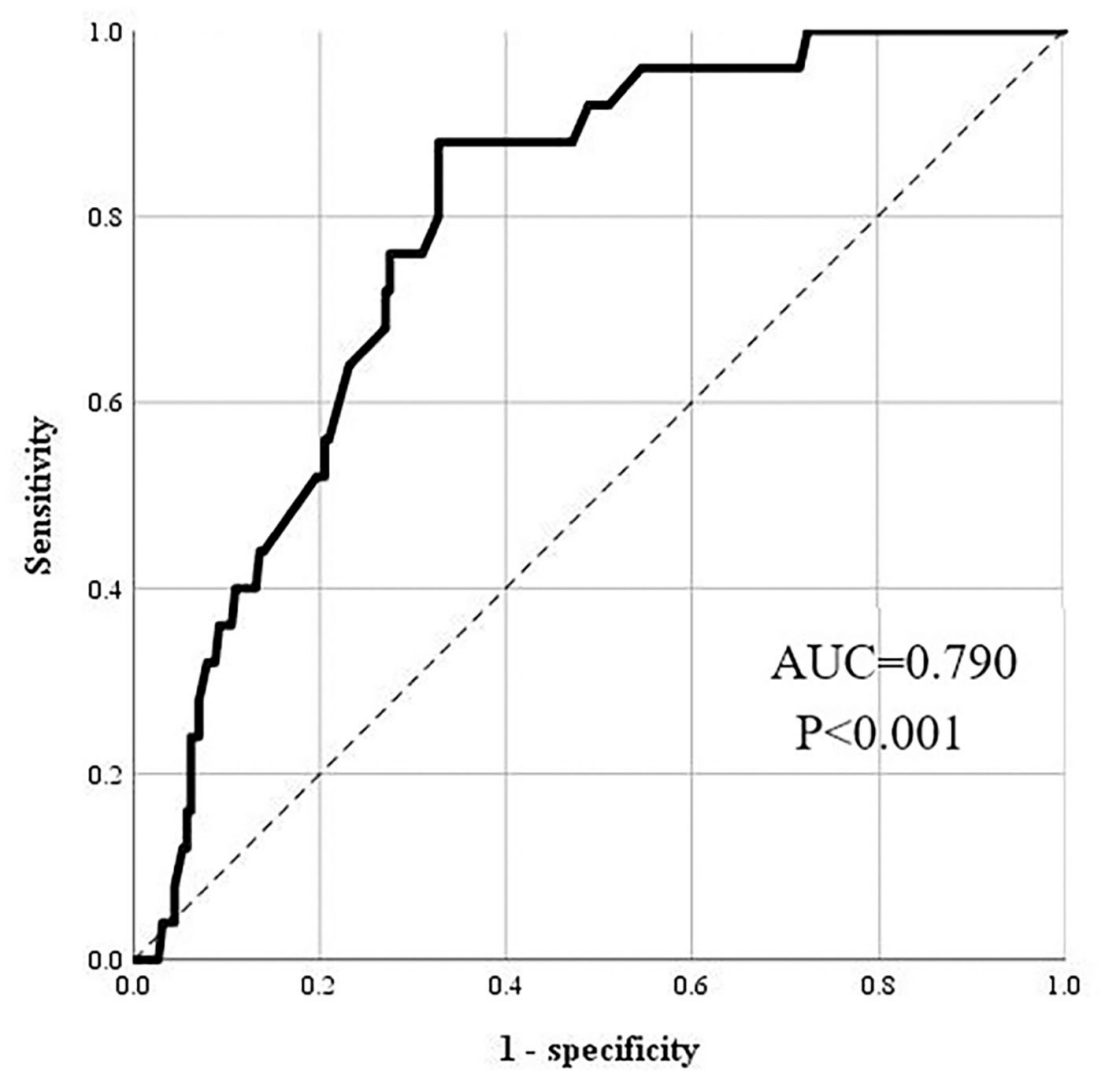
The ROC analysis of the regression model.

After adjusting the mutual effect among variables, the regression model suggested that only LDL was independently associated with the LOS, patients with abnormal LDL were more likely to have long LOS compared with patients with a normal level of LDL (OR = 4.308, 95% CI = 1.385–13.401, *P* = 0.012). No other variable was significantly related to the LOS. Details are seen in Table 2. ROC analysis of the regression model was conducted to evaluate the accuracy of regression model prediction of the LOS. The area under the curve (AUC) was 0.790 (95% CI = 0.714–0.865, *P* < 0.001), meaning good discrimination of the LOS of patients with MDD. Details are shown in Figure 2.

**Table 2:** Binominal logistic regression analysis of the LOS among patients with major depressive disorder.

**Variables**	**B**	**Wald**	**OR (95%CI)**	***P***
**Demographic variables**				
Gender				
Male (reference: Female)	−0.860	2.475	0.423 (0.145–1.235)	0.116
Age				
<18 (reference: ≥18)	0.402	0.366	1.495 (0.406–5.500)	0.545
Job status				
Unemployed (reference: Employed)	0.236	0.230	1.267 (0.482–3.330)	0.632
Marriage status				
Single, separated, divorced or widowed (reference: Married)	−0.329	0.265	0.720 (0.206–2.517)	0.607
Nationality				
Ethnic Han (reference: Ethnic minority)	−1.311	1.015	0.270 (0.021–3.452)	0.314
**Clinical variables**				
Frequency of the previous admission				
0 (reference: ≥1)	0.407	0.327	1.502 (0.372–6.068)	0.568
Category of anti-depressant drugs				
SSRI (reference: SNRI)	−0.962	3.278	0.382 (0.135–1.083)	0.070
**Biochemical variables**				
*Thyroid function*				
Thyroid Stimulating Hormone (TSH)				
Abnormal (reference: Normal)	−0.833	0.581	0.435 (0.051–3.706)	0.446
*Lipoprotein Cholesterol*				
High-density lipoprotein cholesterol (HDL-C)				
Abnormal (reference: Normal)	0.939	3.347	2.557 (0.935–6.989)	0.067
Low-density Lipoprotein cholesterol (LDL-C)				
Abnormal (reference: Normal)	1.461	6.363	4.308 (1.385–13.401)	0.012

## Discussion

This retrospective observational study examined the factors associated with LOS of patients with a specific mental disorder, MDD, from various fields (demographic, clinical, and biochemical). Several aspects of our results merit further discussion, including both statistically significant and non-statistically significant factors.

Low-density lipoprotein was independently associated with the LOS of patients with MDD, for the statistically significant variable. LDL was widely thought to be related to depression from different academic perspectives. Basic medical studies revealed that the cholesterol pathway might play as an important role in the development of depression in animal models; indicating that cerebral lipid metabolism was associated with depression-like behavior in the mouse depression models (53). The clinical intervention studies also indicated that a synergistic treatment of atorvastatin (54) and low-dose omega-3 fatty acids (55), which controlled the level of LDL, could alleviate depressive symptoms among patients with MDD. According to a recent observational study, patients with depression had higher levels of LDL, and the severity of depression was positively related to LDL (56). Similarly, the course of depression was demonstrated to be related to the level of LDL by other studies (57, 58). In other words, abnormal LDL was a risk factor for long-term persistent depression. Our study, which is a real-world non-interventional study rather than a randomized controlled trial (RCT), provided evidence of the relationship between LDL and the LOS of patients with MDD in China that was consistent with previous findings. Furthermore, from the standpoint of in-patient management and alleviating medical resource shortages, predicting the LOS of patients with MDD based on their LDL level measured at the time of admission is a practical strategy that could serve as an important reference for subsequent in-patient beds distribution and adjustment of treatment.

Our results indicated that the category of anti-depressant drugs was not an independent factor with statistical significance related to the LOS among patients with MDD. Although a previous study demonstrated that SNRI, a dual-action anti-depressant drugs, might have a faster onset time than SSRI (59), the SNRI group did not mean shorter LOS compared with the SSRI group under the same discharge requirement criterion from the same hospital in our study. This inconsistency might be caused by the difference in depression severity of the sample at the beginning of admission between the SSRI group and SNRI group. Besides, various tolerance of the adverse effect brought by the anti-depressant drugs might also affect the LOS, as there was some diversity of the side effect (high-risk population, affected system, manifestation, duration, etc.) between these two kinds of anti-depressants (60). However, our results could still suggest that a shorter LOS of patients using SNRI, brought by its quickness and efficacy advantage, than that of patients using SSRI seemed not to exist in an unrandomized clinical sample in China. As the electronic medical system from which we collected data lacked the quantitative evaluation of the original severity of depression and adverse effects of anti-depressant, it is deemed necessary for future studies to further analyze the influence of anti-depressants on LOS based on the exclusion of the effect of the illness severity and adverse effect.

Marriage status was not a statistically independent factor related to the LOS of patients with MDD in the current study. Previous studies about the effect of marriage on the LOS were also inconsistent. A Swiss study (14) and a United Kingdom-based psychiatric in-patient study (61) suggested that marital status was not associated with LOS. While more studies (19, 24, 62) demonstrated married patients were at lower risk of long LOS than patients without this relationship, it might be explained by the increasing social network and emotional and financial support that came with marriage. Combined to our prior work that marriage was an independent factor related to the LOS among patients with schizophrenia and a 14-year follow-up study conducted in China also demonstrated that marriage had a positive influence on the long-term outcome of patients with schizophrenia, we hypothesized that this positive effect that comes with marriage were more obvious in patients with the psychotic disorder than patients with affective disorder. This discrepancy might be because marriage was a method to achieve social inclusion for patients with schizophrenia, rather than for patients with MDD (63, 64).

## Limitations

Some limitations need to be mentioned as a reference for further studies of the LOS among patients with MDD. At first, as we have mentioned previously, some crucial variables were not included in our study, such as quantifiable psychological measurements of depression symptoms and the evaluation of anti-depressants. Although we have identified this flaw in our research, the electronic medical system did not cover this domain of information records. Second, compulsory admission to the psychiatry department was not involved in our research. As the different effects between compulsory admission and voluntary admission on the LOS of patients with MDD, more research is warranted to find out the factors associated with the LOS among compulsory admission samples. Third, the size of our sample was relatively small, for the verification and generalization of our results, a larger sample is necessary for future studies.

## Conclusion

To the best of our knowledge, our research is the first study to bring forward the factors related to the LOS from various domains, especially biochemical indexes and the effect of drugs, among patients with MDD in China. The multivariate regression model suggested that the LDL was the independent factor related to the LOS of patients with MDD and the category of anti-depressant drugs was not significantly associated with the LOS. As good discrimination accuracy of our model proved by ROC analysis, our results could provide theoretical reference for efficient psychiatry hospitalization management and prioritization of allocating medical resources.

## Data availability statement

The raw data supporting the conclusions of this article will be made available by the authors, without undue reservation.

## Author contributions

PC and LW: data collection, literature review, and manuscript drafting. YZ, LX, GZ, and LZ: managed the ethical review process. WL: manuscript drafting and revision. All authors read and approved the final manuscript.

## Funding

This research was supported by the Natural Science Foundation of Hunan Province, China (No. 2020JJ5844 to LZ), Natural Science Foundation of Hunan Province, China (No. 2018JJ2592 to WL), Hunan Key Research and Development Program (No. 2018SK2136 to WL), and Natural Science Foundation of China (82171518 to LZ).

## Conflict of interest

The authors declare that the research was conducted in the absence of any commercial or financial relationships that could be construed as a potential conflict of interest.

## Publisher's Note

All claims expressed in this article are solely those of the authors and do not necessarily represent those of their affiliated organizations, or those of the publisher, the editors and the reviewers. Any product that may be evaluated in this article, or claim that may be made by its manufacturer, is not guaranteed or endorsed by the publisher.

## References

[1] Global, regional, and national incidence, prevalence, and years lived with disability for 354 diseases and injuries for 195 countries and territories, 1990-2017: 1990-2017: a systematic analysis for the Global Burden of Disease Study 2017. Lancet. (2018) 392:1789–858. 10.1016/S0140-6736(18)32279-7 30496104 PMC6227754

[2] Huang Y Wang Y Wang H Liu Z Yu X Yan J. Prevalence of mental disorders in China: a cross-sectional epidemiological study. Lancet Psychiatry. (2019) 6:211–24. 10.1016/S2215-0366(18)30511-X 30792114

[3] Global burden of 369 diseases and injuries in 204 countries and territories, 1990-2019: 1990-2019: a systematic analysis for the Global Burden of Disease Study (2019). Lancet. (2020) 396:1204–22. 33069326 10.1016/S0140-6736(20)30925-9 PMC7567026

[4] Mitchell A Selmes T. Why don't patients attend their appointments? Maintaining engagement with psychiatric services. Adv Psychiatr Treat. (2007) 13:423–34. 10.1192/apt.bp.106.003202

[5] Bulloch AG Patten SB. Non-adherence with psychotropic medications in the general population. Soc Psychiatry Psychiatr Epidemiol. (2010) 45:47–56. 10.1007/s00127-009-0041-5 19347238

[6] Banerjee S Varma RP. Factors affecting non-adherence among patients diagnosed with unipolar depression in a psychiatric department of a tertiary hospital in Kolkata, India. Depress Res Treat. (2013) 2013:809542. 10.1155/2013/809542 24381752 PMC3868196

[7] Lingam R Scott J. Treatment non-adherence in affective disorders. Acta Psychiatr Scand. (2002) 105:164–72. 10.1034/j.1600-0447.2002.1r084.x 11939969

[8] Eikelenboom M Beekman ATF Penninx B Smit JH. A 6-year longitudinal study of predictors for suicide attempts in major depressive disorder. Psychol Med. (2019) 49:911–21. 10.1017/S0033291718001423 29897037

[9] Vuorilehto M Valtonen HM Melartin T Sokero P Suominen K Isometsä ET. Method of assessment determines prevalence of suicidal ideation among patients with depression. Eur Psychiatry. (2014) 29:338–44. 10.1016/j.eurpsy.2013.08.005 24176645

[10] Li H Luo X Ke X Dai Q Zheng W Zhang C. Major depressive disorder and suicide risk among adult outpatients at several general hospitals in a Chinese Han population. PLoS ONE. (2017) 12:e0186143. 10.1371/journal.pone.0186143 29016669 PMC5634639

[11] Lu J Xu X Huang Y Li T Ma C Xu G. Prevalence of depressive disorders and treatment in China: a cross-sectional epidemiological study. Lancet Psychiatry. (2021) 8:981–90. 10.1016/S2215-0366(21)00251-0 34559991

[12] World Health O. Mental Health Atlas 2017. Geneva: World Health Organization (2018).

[13] Guidelines for the establishment of medical institutions: Chinese National Health Commission; (2022). Available online at: http://www.nhc.gov.cn/yzygj/s3594q/202201/2156670fb665406ea98f9c1a6329954d/files/f527c66af01742928199dc55216e6c8e.pdf

[14] Habermeyer B De Gennaro H Frizi RC Roser P Stulz N. Factors associated with length of stay in a Swiss Mental Hospital. Psychiatr Q. (2018) 89:667–74. 10.1007/s11126-018-9569-4 29430589

[15] Addisu F Wondafrash M Chemali Z Dejene T Tesfaye M. Length of stay of psychiatric admissions in a general hospital in Ethiopia: a retrospective study. Int J Ment Health Syst. (2015) 9:13. 10.1186/s13033-015-0006-x 25780386 PMC4361196

[16] Fugger G Waldhör T Hinterbuchinger B Pruckner N König D Gmeiner A. Pattern of inpatient care for depression: an analysis of 232,289 admissions. BMC Psychiatry. (2020) 20:375. 10.1186/s12888-020-02781-z 32677945 PMC7364660

[17] Lee DY Park J Noh JS Roh HW Ha JH Lee EY. Characteristics of dimensional psychopathology in suicidal patients with major psychiatric disorders and its association with the length of hospital stay: algorithm validation study. JMIR Ment Health. (2021) 8:e30827. 10.2196/30827 34477555 PMC8449292

[18] Oladeji BD Ogundele AT Dairo M. Determinants of length of stay in the psychiatric wards of the University College Hospital, Ibadan, Nigeria. Afr J Med Med Sci. (2012) 41:147–52. 23185912

[19] Bruce M Smith J. Length of stay among multi-ethnic psychiatric in-patients in the United Kingdom. Compr Psychiatry. (2020) 102:152201. 10.1016/j.comppsych.2020.152201 32898735

[20] Gopalakrishna G Ithman M Malwitz K. Predictors of length of stay in a psychiatric hospital. Int J Psychiatry Clin Pract. (2015) 19:238–44. 10.3109/13651501.2015.1062522 26073671

[21] Noohi S Kalantari S Hasanvandi S Elikaei M. Determinants of length of stay in a psychiatric ward: a retrospective chart review. Psychiatr Q. (2020) 91:273–87. 10.1007/s11126-019-09699-0 31865511

[22] Choy L Dunn E. Determinants of length of stay in a general hospital psychiatric unit in Hong Kong. Hong Kong J Psychiatry. (2007) 17:131–9.

[23] Ismail Z Arenovich T Grieve C Willett P Sajeev G Mamo DC. Predicting hospital length of stay for geriatric patients with mood disorders. Can J Psychiatry. (2012) 57:696–703. 10.1177/070674371205701008 23149285

[24] Masters GA Baldessarini RJ Öngür D Centorrino F. Factors associated with length of psychiatric hospitalization. Compr Psychiatry. (2014) 55:681–7. 10.1016/j.comppsych.2013.11.004 24387922

[25] Yang F Yang BX Stone TE Wang XQ Zhou Y Zhang J. Stigma toward depression in a community-based sample in China. Compr Psychiatry. (2020) 97:152152. 10.1016/j.comppsych.2019.152152 31838297

[26] Chen H Phillips M Cheng H Chen Q Chen X Fralick D. Mental health law of the people's Republic of China (English translation with annotations): translated and annotated version of China's new Mental Health Law. Shanghai Arch Psychiatry. (2012) 24:305–21. 10.3969/j.issn.1002-0829.2012.06.001 25324635 PMC4198897

[27] Demartini B Ranieri R Masu A Selle V Scarone S Gambini O. Depressive symptoms and major depressive disorder in patients affected by subclinical hypothyroidism: a cross-sectional study. J Nerv Ment Dis. (2014) 202:603–7. 10.1097/NMD.0000000000000168 25010109

[28] Park YJ Lee EJ Lee YJ Choi SH Park JH Lee SB. Subclinical hypothyroidism (SCH) is not associated with metabolic derangement, cognitive impairment, depression or poor quality of life (QoL) in elderly subjects. Arch Gerontol Geriatr. (2010) 50:e68–73. 10.1016/j.archger.2009.05.015 19545916

[29] Brouwer JP Appelhof BC Hoogendijk WJ Huyser J Endert E Zuketto C. Thyroid and adrenal axis in major depression: a controlled study in outpatients. Eur J Endocrinol. (2005) 152:185–91. 10.1530/eje.1.01828 15745924

[30] Loh HH Lim LL Yee A Loh HS. Association between subclinical hypothyroidism and depression: an updated systematic review and meta-analysis. BMC Psychiatry. (2019) 19:12. 10.1186/s12888-018-2006-2 30621645 PMC6325749

[31] Bauer M Goetz T Glenn T Whybrow PC. The thyroid-brain interaction in thyroid disorders and mood disorders. J Neuroendocrinol. (2008) 20:1101–14. 10.1111/j.1365-2826.2008.01774.x 18673409

[32] Berlin I Corruble E. Thyroid hormones and anti-depressant response. Am J Psychiatry. (2002) 159:1441. 10.1176/appi.ajp.159.8.1441 12153857

[33] Abulseoud OA Gitlin M Altshuler L Frye MA. Baseline thyroid indices and the subsequent response to citalopram treatment, a pilot study. Brain Behav. (2013) 3:89–94. 10.1002/brb3.109 23533025 PMC3607150

[34] Woolf PD Nichols D Porsteinsson A Boulay R. Thyroid evaluation of hospitalized psychiatric patients: the role of TSH screening for thyroid dysfunction. Thyroid. (1996) 6:451–6. 10.1089/thy.1996.6.451 8936670

[35] Sjögren B Svenningsson P. Depletion of the lipid raft constituents, sphingomyelin and ganglioside, decreases serotonin binding at human 5-HT7(a) receptors in HeLa cells. Acta Physiol. (2007) 190:47–53. 10.1111/j.1365-201X.2007.01687.x 17428232

[36] Freemantle E Chen GG Cruceanu C Mechawar N Turecki G. Analysis of oxysterols and cholesterol in prefrontal cortex of suicides. Int J Neuropsychopharmacol. (2013) 16:1241–9. 10.1017/S1461145712001587 23369504

[37] Blier P. Neurobiology of depression and mechanism of action of depression treatments. J Clin Psychiatry. (2016) 77:e319. 10.4088/JCP.13097tx3c 27046319

[38] Lee HJ Kim YK. Serum lipid levels and suicide attempts. Acta Psychiatr Scand. (2003) 108:215–21. 10.1034/j.1600-0447.2003.00115.x 12890277

[39] Li H Zhang X Sun Q Zou R Li Z Liu S. Association between serum lipid concentrations and attempted suicide in patients with major depressive disorder: a meta-analysis. PLoS ONE. (2020) 15:e0243847. 10.1371/journal.pone.0243847 33301469 PMC7728216

[40] Stephens RJ White SE Cudnik M Patterson ES. Factors associated with longer length of stay for mental health emergency department patients. J Emerg Med. (2014) 47:412–9. 10.1016/j.jemermed.2014.04.040 25074781

[41] Rang HP Dale MM Ritter JM Flower RJ. Other Peripheral Mediators: 5-Hydroxytryptamine and Purines. (2007). p. 189–201.

[42] Rominger A Cumming P Brendel M Xiong G Zach C Karch S. Altered serotonin and dopamine transporter availabilities in brain of depressed patients upon treatment with escitalopram: a [123 I]β-CIT SPECT study. Eur Neuropsychopharmacol. (2015) 25:873–81. 10.1016/j.euroneuro.2014.12.010 25819144

[43] Stahl SM. Essential Psychopharmacology: Neuroscientific Basis and Practical Applications. Cambridge: Cambridge university press (2000).

[44] Nutt DJ. Relationship of neurotransmitters to the symptoms of major depressive disorder. J Clin Psychiatry. (2008) 69:4–7. 18494537

[45] Hirschfeld RM Mallinckrodt C Lee TC Detke MJ. Time course of depression-symptom improvement during treatment with duloxetine. Depress Anxiety. (2005) 21:170–7. 10.1002/da.20071 16035056

[46] Nierenberg AA Greist JH Mallinckrodt CH Prakash A Sambunaris A Tollefson GD. Duloxetine versus escitalopram and placebo in the treatment of patients with major depressive disorder: onset of anti-depressant action, a non-inferiority study. Curr Med Res Opin. (2007) 23:401–16. 10.1185/030079906X167453 17288694

[47] Mlinar B Montalbano A Baccini G Tatini F Berlinguer Palmini R Corradetti R. Nonexocytotic serotonin release tonically suppresses serotonergic neuron activity. J Gen Physiol. (2015) 145:225–51. 10.1085/jgp.201411330 25712017 PMC4338157

[48] Celada P Puig M Amargós-Bosch M Adell A Artigas F. The therapeutic role of 5-HT1A and 5-HT2A receptors in depression. J Psychiatry Neurosci. (2004) 29:252–65. 15309042 PMC446220

[49] Thase ME Entsuah AR Rudolph RL. Remission rates during treatment with venlafaxine or selective serotonin reuptake inhibitors. Br J Psychiatry. (2001) 178:234–41. 10.1192/bjp.178.3.234 11230034

[50] Papakostas GI Thase ME Fava M Nelson JC Shelton RC. Are anti-depressant drugs that combine serotonergic and noradrenergic mechanisms of action more effective than the selective serotonin reuptake inhibitors in treating major depressive disorder? A meta-analysis of studies of newer agents. Biol Psychiatry. (2007) 62:1217–27. 10.1016/j.biopsych.2007.03.027 17588546

[51] Diagnostic Diagnostic and Statistical Manual of Mental Disorders 5th Edn. Arlington, VA: American Psychiatric Association (2013).

[52] Barnett P Mackay E Matthews H Gate R Greenwood H Ariyo K. Ethnic variations in compulsory detention under the Mental Health Act: a systematic review and meta-analysis of international data. Lancet Psychiatry. (2019) 6:305–17. 10.1016/S2215-0366(19)30027-6 30846354 PMC6494977

[53] Gulbins E Palmada M Reichel M Lüth A Böhmer C Amato D. Acid sphingomyelinase-ceramide system mediates effects of antidepressant drugs. Nat Med. (2013) 19:934–8. 10.1038/nm.3214 23770692

[54] Haghighi M Khodakarami S Jahangard L Ahmadpanah M Bajoghli H Holsboer-Trachsler E. In a randomized, double-blind clinical trial, adjuvant atorvastatin improved symptoms of depression and blood lipid values in patients suffering from severe major depressive disorder. J Psychiatr Res. (2014) 58:109–14. 10.1016/j.jpsychires.2014.07.018 25130678

[55] Tajalizadekhoob Y Sharifi F Fakhrzadeh H Mirarefin M Ghaderpanahi M Badamchizade Z. The effect of low-dose omega 3 fatty acids on the treatment of mild to moderate depression in the elderly: a double-blind, randomized, placebo-controlled study. Eur Arch Psychiatry Clin Neurosci. (2011) 261:539–49. 10.1007/s00406-011-0191-9 21318452

[56] Wagner CJ Musenbichler C Böhm L Färber K Fischer AI von Nippold F. LDL cholesterol relates to depression, its severity, and the prospective course. Prog Neuropsychopharmacol Biol Psychiatry. (2019) 92:405–11. 10.1016/j.pnpbp.2019.01.010 30779936

[57] Lehto SM Niskanen L Tolmunen T Hintikka J Viinamäki H Heiskanen T. Low serum HDL-cholesterol levels are associated with long symptom duration in patients with major depressive disorder. Psychiatry Clin Neurosci. (2010) 64:279–83. 10.1111/j.1440-1819.2010.02079.x 20374538

[58] Lehto SM Hintikka J Niskanen L Tolmunen T Koivumaa-Honkanen H Honkalampi K. Low HDL cholesterol associates with major depression in a sample with a 7-year history of depressive symptoms. Prog Neuropsychopharmacol Biol Psychiatry. (2008) 32:1557–61. 10.1016/j.pnpbp.2008.05.021 18583011

[59] Nierenberg AA. Do some anti-depressants work faster than others? J Clin Psychiatry. (2001) 62:22–5. 11444763

[60] Carvalho AF Sharma MS Brunoni AR Vieta E Fava GA. The safety, tolerability and risks associated with the use of newer generation antidepressant drugs: a critical review of the literature. Psychother Psychosom. (2016) 85:270–88. 10.1159/000447034 27508501

[61] Newman L Harris V Evans LJ Beck A. Factors associated with length of stay in psychiatric inpatient services in London, UK. Psychiatr Q. (2018) 89:33–43. 10.1007/s11126-017-9498-7 28367585 PMC5807484

[62] Pauselli L Verdolini N Bernardini F Compton MT Quartesan R. Predictors of length of stay in an inpatient psychiatric unit of a general hospital in Perugia, Italy. Psychiatr Q. (2017) 88:129–40. 10.1007/s11126-016-9440-4 27167133

[63] Baumgartner JN Susser E. Social integration in global mental health: what is it and how can it be measured? Epidemiol Psychiatr Sci. (2013) 22:29–37. 10.1017/S2045796012000303 22794167 PMC3733104

[64] Yang LH Chen FP Sia KJ Lam J Lam K Ngo H. “What matters most:” a cultural mechanism moderating structural vulnerability and moral experience of mental illness stigma. Soc Sci Med. (2014) 103:84–93. 10.1016/j.socscimed.2013.09.009 24507914

